# Effect of Aprotinin and Avifavir^®^ Combination Therapy for Moderate COVID-19 Patients

**DOI:** 10.3390/v13071253

**Published:** 2021-06-27

**Authors:** Andrey A. Ivashchenko, Valeria N. Azarova, Alina N. Egorova, Ruben N. Karapetian, Dmitry V. Kravchenko, Natalia V. Krivonos, Vladimir G. Loginov, Stanislav V. Poyarkov, Elena A. Merkulova, Olga S. Rosinkova, Nikolay P. Savchuk, Mikhail A. Topr, Elena N. Simakina, Elena V. Yakubova, Alexandre V. Ivachtchenko

**Affiliations:** 1 ChemRar High-Tech Center, 141401 Moscow, Russia; ai@chemrar.ru (A.A.I.); av@chemdiv.com (A.V.I.); 2 IPHARMA LLC, Skolkovo Innovative Centre, 121205 Moscow, Russia; avn@ipharma.ru (V.N.A.); ean@ipharma.ru (A.N.E.); nvk@ipharma.ru (N.V.K.); eam@ipharma.ru (E.A.M.); 3 ChemRar Research Institute, 141401 Moscow, Russia; 4 Chemical Diversity Research Institute, 141401 Moscow, Russia; dk@chemrar.ru (D.V.K.); nsavchuk@chemdiv.com (N.P.S.); 5 JSC “Special Economic Zones”, 125009 Moscow, Russia; vldqru@me.com; 6 Centre for Strategic Planning of the Federal Medical-Biological Agency (FMBA), 119121 Moscow, Russia; poyarkov.stanislav@gmail.com; 7 Clinical Hospital No. 1, 214006 Smolensk, Russia; o.peregontseva80@mail.ru (O.S.R.); e.simakina@mail.ru (E.N.S.); 8 ChemDiv Inc., San Diego, CA 92121-3103, USA; mtopr@mfm-ny.com; 9 Chromis LLC, Skolkovo Innovative Centre, 121205 Moscow, Russia; ey@chemrar.ru

**Keywords:** COVID-19, SARS-CoV-2, aprotinin, favipiravir

## Abstract

COVID-19 is a contagious multisystem inflammatory disease caused by a severe acute respiratory syndrome coronavirus 2 (SARS-CoV-2). We studied the efficacy of Aprotinin (nonspecific serine proteases inhibitor) in combination with Avifavir^®^ or Hydroxychloroquine (HCQ) drugs, which are recommended by the Russian Ministry of Health for the treatment therapy of moderate COVID-19 patients. This prospective single-center study included participants with moderate COVID-19-related pneumonia, laboratory-confirmed SARS-CoV-2, and admitted to the hospitals. Patients received combinations of intravenous (IV) Aprotinin (1,000,000 KIU daily, 3 days) and HCQ (cohort 1), inhalation (inh) treatment with Aprotinin (625 KIU four times per day, 5 days) and HCQ (cohort 2) or IV Aprotinin (1,000,000 KIU daily for 5 days) and Avifavir (cohort 3). In cohorts 1–3, the combination therapy showed 100% efficacy in preventing the transfer of patients (*n* = 30) to the intensive care unit (ICU). The effect of the combination therapy in cohort 3 was the most prominent, and the median time to SARS-CoV-2 elimination was 3.5 days (IQR 3.0–4.0), normalization of the CRP concentration was 3.5 days (IQR 3–5), of the D-dimer concentration was 5 days (IQR 4 to 5); body temperature was 1 day (IQR 1–3), improvement in clinical status or discharge from the hospital was 5 days (IQR 5–5), and improvement in lung lesions of patients on 14 day was 100%.

## 1. Introduction

SARS-CoV-2 spread globally, and in March 2021, the total number of infections in the world exceeded 123 million, with more than 2.7 million deaths [1]. Given that COVID-19 poses a serious threat to public health and the economy around the world, an urgent need exists for a new effective drugs for the treatment and prophylaxis of SARS-CoV-2 infections. The SARS-CoV-2 replication inhibitor favipiravir (Avifavir^®^) [2,3], together with Hydroxychloroquine, umifenovir, and lopinavir+ritonavir, were among the first drugs repurposed and recommended by the Russian Ministry of Health for the treatment of COVID-19 [4].

Previously, we demonstrated the efficacy of Avifavir^®^ in the treatment of patients with moderate COVID-19 [3]. However, taking into account the complex nature of the SARS-CoV-2 pathogenesis and multiorgan involvement, a combination of direct virus-acting and host-targeted drugs could be clinically beneficial for the therapy of COVID-19. One of the promising drug candidates for the combination therapy of COVID-19 is aprotinin, a natural protease inhibitor with a long history of clinical use since the 1960s, a good safety profile, and anti-inflammatory activity [5,6,7]. Recently, it was demonstrated that aprotinin inhibits transmembrane serine protease 2 (TMPRSS2), a host cell protease responsible for the cleavage and activation of the spike protein of SARS-CoV-2 [8,9], and downregulates cellular proteases during replication cycles [10]. Thus, in addition to an anti-inflammatory effect, it is suggested that aprotinin can prevent SARS-CoV-2 penetration into susceptible cells and inhibits its replication. Our preliminary case series demonstrated a good potential of aprotinin for prevention [11] and as a part of combination therapy for the treatment [12] of COVID-19.

Therefore, we evaluated the efficacy of aprotinin in combination with drugs recommended by the Russian Ministry of Health for the treatment of COVID-19: HCQ and Avifavir^®^. Here, we report the results of a pilot noncomparative clinical study of the efficacy and safety of a combination therapy of aprotinin with Avifavir^®^ or HCQ for moderate COVID-19 patients.

## 2. Materials and Methods

### 2.1. Ethics

This study was conducted at the Smolensk Clinical Hospital, Russia from June (11 June 2020—1st patient was included) to August 2020. The COVID-19-aprotinin-01 study protocol and the amendment to the protocol were approved by the Independent Ethics Committee of Smolensk Clinical Hospital (protocols NO. 38 from 2 June and 2 July 2020, respectively) and registered at the U.S. National Library of Medicine (NCT04527133). All patients participated in this study provided their written informed consent. The informed consent form was approved by a local ethics committee (Independent Ethics Committee of Smolensk Clinical Hospital #1) before the study was started at the research site.

### 2.2. Study Design and Patients

This was an open noncomparative study of the safety and efficacy of aprotinin on the patients hospitalized with COVID-19. Characteristics of the patients and exclusion criteria are presented in the Appendix A.

Participants were divided into 3 cohorts of 10 patients in each: cohort 1—combination of aprotinin (IV) (Gordox^®^ 1,000,000 KIU daily, 3 days), HCQ (400/200 mg, twice a day, 5 to 6 days), and standard of care (SOC); cohort 2—combinations of inhaled aprotinin (Gordox^®^ 625 KIU four times per day, 5 days), HCQ (400/200 mg, twice a day, 5 to 6 days), and SOC; and cohort 3—combinations of aprotinin (IV) (Gordox^®^ 1,000,000 KIU daily, 5 days), oral Avifavir^®^ (2000 mg twice on the first day, then 800 mg twice a day, 10 days), and SOC. Patients in cohorts 1–3 received thromboembolic prophylaxis with an anticoagulant enoxaparin (40 mg, once a day, 14 to 15 days). Patients with a score of 4 on the WHO-OSCI had supportive oxygen therapy via nasal cannula or face mask. None of the patients had invasive or NIV mechanical ventilation at the baseline.

### 2.3. Efficacy Endpoints

The primary efficacy endpoint of the study was time to normalization of the following parameters: elimination of SARS-CoV-2 (defined as two negative results from a RT-PCR assay with at least a 24-h interval), CRP, and D-dimer concentrations.

Key secondary clinical endpoints were: time to body temperature normalization (<37 °C) and changes from baseline of the laboratory parameters during 14 days, which included hematology: CRP values and coagulogram; changes from the baseline of lung parenchyma on a tomography chest CT scan on days 7 and 14; frequency of the clinical status improvement by 2 scores in accordance with the WHO Ordinal scale of clinical improvement (WHO-OSCI) or discharge from the hospital before day 14; frequency of transfer to the ICU, frequency of the NIV, and frequency of the invasive ventilation; mortality rate; and frequency of adverse events and serious adverse events of various severities according to subjective complaints, physical examination, vital signs, laboratory tests, and electrocardiogram.

### 2.4. Procedures

Clinical manifestations, including persistent fever >38 °C, respiratory rate, oxygen saturation, and oxygen therapy requirement, and biological parameters, including CRP, D-dimer, neutrophil, lymphocyte and platelet counts, INR, prothrombin, and fibrinogen, were recorded at the baseline and at discharge from the hospital. The median time to improve the clinical state by 2 points was determined according to the WHO-OSCI. Chest CT was done with a single inspiratory phase with patients in the supine position. Radiologists classified the CT scan as typical, equivocal, or negative for COVID-19 and described the main CT features: ground glass opacity, crazy-paving pattern, and consolidation. A semi-quantitative scoring system was used to estimate the pulmonary involvement of the observed abnormalities based on the area involved: mild (<25%), moderate (25–50%), severe (51–75%), or diffuse (>75%) [13].

### 2.5. Statistical Analysis

The sample size was based on the exact single-stage Phase II assessment at one-sided α = 0.05 and 80% power [14]. Continuous variables with a normal distribution were expressed as the mean (SD) and with a non-normal distribution as the median with interquartile range (IQR) and compared using a 2-tailed, paired *t*-test for parametric data and Wilcoxon rank-sum test for nonparametric data. The categorical variables were presented as the absolute and relative (in percentage) frequencies and compared using a chi-square test. The efficacy endpoints (time to viral clearance, time to CRP normalization (≤10 mg/L), time to D-dimer normalization (<253 ng/mL), time to temperature normalization (<37 °C), and time to improvement in clinical status) were estimated using Kaplan–Meier curves. For groups, a comparison log-rank test was used (*p*-value ˂ 0.05 was considered significant).

## 3. Results

The cohorts were generally comparable, while some differences existed; the proportions of males and females were higher in cohort 3 as compared to cohorts 1 and 2, and some of the patients in cohort 1 had a score of 3 (according to the WHO-OSCI), while all the patients in cohorts 2 and 3 had a score of 4 (requiring oxygen therapy; Appendix A).

**Figure 1:**
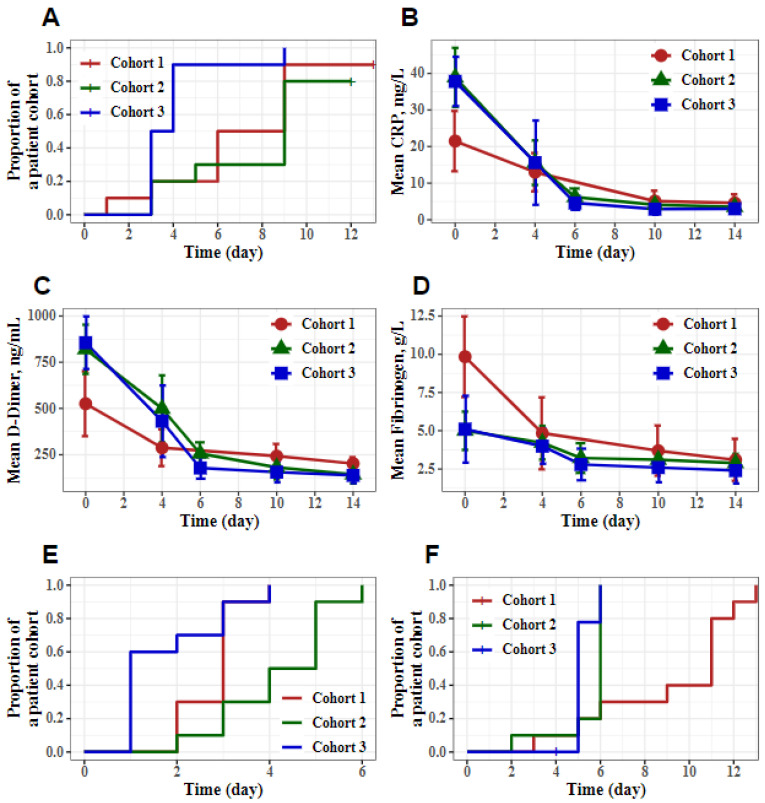
Effect of aprotinin and HCQ or Avifavir^®^ (cohorts 1–3) combination therapy on the primary and secondary efficacy endpoints in COVID-19 patients. The panels show (**A**) the elimination of SARS-CoV-2 (days) and (**B**) CRP concentration (mg/L). Normal concentration of CRP is ≤ 10 mg/L. (**C**) D-dimer concentration (ng/mL). Normal concentration of D-dimer is ≤253 ng/mL. (**D**) Fibrinogen concentration (g/L). Normal range of fibrinogen is 2–4 g/L. (**E**) Normalization of the body temperatures (<37 °C) time since the initiation of treatment (days). (**F**) Improvement of the clinical state by 2 points (according to the WHO-OSCI) in the time since the initiation of treatment (days). (A,E,F) Show the time since the initiation of treatment (days). (B,C,D) Show the time from inclusion (days).

An analysis of the primary and secondary efficacy points revealed that combination therapy with aprotinin (IV) + Avifavir in association with SOC was beneficial for COVID-19 patients (Table 1). In particular, the median time to SARS-CoV-2 elimination was 3.5 (IQR 3–4) days for cohort 3 and 7.5 (IQR 6–9) and 9 (IQR 5–9) days for cohorts 1 and 2, respectively (the difference was significant, *p* = 0.019 and *p* = 0.006 as compared to patients from cohorts 3, Figure 1A). The median time to CRP normalization was 3.5 (IQR 3–5) days for cohort 3 and 6 (IQR 6–6) and 4 (IQR 3–5) days for cohorts 1 and 2, respectively. The difference in this parameter between cohorts 1 and 3 was significant (*p* < 0.001, Figure 1B).

The efficacy of the aprotinin combinations on the normalization of thrombosis markers (D-dimer and fibrinogen) in the patients’ blood are presented in Figure 1C,D. The increased D-dimer levels quickly returned to normal values with a median of 4.5 (IQR 3–6), 9 (IQR 5–9), and 5 (IQR 4–5) days for cohorts 1, 2, and 3, respectively (Figure 1C). The difference in this parameter between cohorts 2 and 3 was significant (*p* = 0.002). The elevated baseline fibrinogen levels returned to normal values on day 4, presumably as a result of the therapy (Figure 1D).

The dynamics of the INR and prothrombin changes were used to monitor blood-thinning anticoagulants and to check blood-clotting problems. Both the INR and Quick prothrombin tests were defined as normal at admission and discharge of the patients from the hospital. The patients presented normal values for neutrophils and leukocytes when admitted to the hospital and when discharged from the hospital after the aprotinin combination therapy.

The median time to normalization of the body temperatures of the patients in cohorts 1–3 was 3 (IQR 2–3), 4.5 (IQR 3–5), and 1 (IQR 1–3) days, respectively. The difference in this parameter between cohorts 2 and 3 was significant (*p* < 0.001). The median time to improve the clinical state by two points was 11 (IQR 6–11), 6 (IQR 6–6), and 5 (IQR 5–5) days for cohorts 1–3, respectively (Figure 1E,F). This parameter differed significantly between cohorts 1 and 2 and from that in cohort 3 (*p* = 0.004 and *p* = 0.036, respectively).

Importantly, none of the participants in cohorts 1–3 of the administered aprotinin combinations were transferred to the ICU for ALV or NIV. All the patients in cohorts 1–3 were discharged from the hospital, and no adverse events were recorded.

For the retrospective comparison, our results from two historical cohorts with COVID-19 patients treated with Avifavir^®^ + SOC (*n* = 40, cohort 4) or HCQ + SOC (*n* = 20, cohort 5) were added to Table 1 (the study was conducted in six Russian hospitals from 27 April to 4 July 2020) [3].

## 4. Discussion

In this study, we evaluated the efficacy of aprotinin in combination with HCQ and Avifavir^®^ in patients admitted to the hospital due to COVID-19-associated pneumonia. HCQ and Avifavir^®^ are recommended by the Russian Ministry of Health for the treatment of the new coronavirus infection COVID-19 [4]. Retrospectively, we compared these results with our data from the Phase II/III clinical trials of Avifavir^®^ among hospitalized patients with moderate COVID-19 pneumonia [3]. It is important to mention that, while HCQ was recommended for the therapy of COVID-19 patients in Russia at the time when this study was conducted, later results demonstrated that it does not improve the clinical status of the patients hospitalized with COVIV-19 compared to the standard care [15,16]. It allowed us to consider HCQ+SOC as SOC and helped us to better interpret the effects of aprotinin by comparing the median time to SARS-CoV-2 elimination in cohorts 1 and 2 vs. 5; we could see that, at the studied doses, aprotinin did not affect the replication of SARS-CoV-2, but it significantly reduced the level of CRP (comparing the median time to CRP normalization in cohorts 1 and 2 vs. 5 and cohort 3 vs. 4), which was in agreement with its known anti-inflammatory activity [17].

As we hypothesized, the most significant results were demonstrated with a combination of aprotinin and Avifavir^®^. It reduced the time to normalization of the CRP and D-dimer concentrations in the patients’ blood and overall improved the clinical outcome and the median time to SARS-CoV-2 elimination, and the body temperature normalization was shorter compared to cohorts 1 and 2; the elevated fibrinogen levels returned to normal concentrations on day 4. As it was previously demonstrated, Avifavir^®^ itself enabled SARS-CoV-2 viral clearance in 62.5% of patients within 4 days of therapy but had little effect on the concentration of CRP, which is a marker of the severity of COVID-19 [3]. Taking into account the results from our historical cohorts, we can hypothesize that the better median time to SARS-CoV-2 elimination in cohort 3 was due to the effect of Avifavir^®^, but the improved recovery from the infection was most likely due to the actions of aprotinin.

Despite the limitations of this pilot clinical study, such as the low number of patients per cohort and absence of prospective cohorts with aprotinin+SOC, Avifavir^®^+SOC, and SOC, this clinical study revealed, for the first time, the potency of aprotinin combination therapy for patients hospitalized with moderate COVID-19 pneumonia and requiring oxygen therapy. Significantly, none of the patients in cohorts 1–3 treated with the aprotinin combinations were transferred to the ICU for ALV or NIV, no adverse events were recorded, and all the patients were discharged from the hospital.

Taken together, these results can be considered as a promising first step in the evaluation of aprotinin and open the possibility for the initiation of a multicenter, randomized trial of combination aprotinin therapy in patients with moderate and severe COVID-19.

## 5. Conclusions

Our findings demonstrates that therapy with a combination of aprotinin with Avifavir^®^ showed promising results in preventing disease progression in patients hospitalized with COVID-19-associated pneumonia and requiring oxygen therapy, as none of the patients were transferred to the ICU for mechanical ventilation or noninvasive ventilation, and their hospital stays were shortened.

## Figures and Tables

**Table 1:** Primary and secondary efficacy endpoints.

Primary and Secondary Efficacy Endpoints	Cohort 1 (Aprotinin IV + HCQ + SOC)	Cohort 2 (Aprotinin inh + HCQ + SOC)	Cohort 3 (Aprotinin IV+Avifavir^®^+SOC)	Cohort 4 [3] (Avifavir^®^ + SOC)	Cohort 5 [3] (HCQ + SOC)
Primary efficacy endpoints, * *p*					
Median time to elimination of SARS-CoV-2 confirmed by RT-PCR, days (IQR)	7.5 (6–9), *p =* 0.019	9.0 (5–9), *p *= 0.006	3.5 (3–4)	4.5 (4–9)	9.0 (5–9)
Median time to normalization of CRP concentration (≤10 mg/L) in patient’s blood, days (IQR)	6.0 (6–6), *p* < 0.001	4.0 (3–5), *p *= 0.821	3.5 (3–5)	14.0 (5.5–14)	14.0 (14–14)
Median time to normalization of D-dimer concentration (<253 ng/mL) in patient’s blood, days (IQR)	4.5 (3–6), *p *= 0.675	9.0 (5–9), *p *= 0.002	5.0 (4–5)	NA	NA
Secondary efficacy endpoints, * *p*					
Median time to normalization of body temperature (<37 °C), days (IQR)	3.0 (2–3), *p* = 0.090	4.5 (3–5), *p* < 0.001	1.0 (1–3)	2.0 (1–3)	4.0 (1–8)
Median time to improvement in clinical status by 2 points on the WHO-OSCI, days (IQR)	11.0 (6–11), *p *= 0.004	6.0 (6–6), *p *= 0.036	5.0 (5–5)	14.0 (11.5–16)	13.0 (11.5–15.5)
Changes in lung lesions according to chest CT data on day 14 after hospitalization					
Improvement, no. (%)	6 (60)	10 (100)	10 (100)	36 (90)	16 (80)
Without changes, no. (%)	4 (40)	0	0	2 (5)	2 (10)
Worsening, no. (%)	0	0	0	2 (5)	2 (10

* *p* < 0.05, compared between efficacy endpoints in patients from cohorts 1 and 2 and those in patients from cohort 3 (aprotinin (IV) + Avifavir + SOC) using a log-rank test. NA–data not applicable.

## Data Availability

The datasets used and/or analyzed during the current study are available from the corresponding author upon reasonable request.

## Appendix A

**Table A1:** Demographic and baseline characteristics of the patients.

Characteristic	Cohort 1	Cohort 2	Cohort 3
Age			
Years, mean (SD)	44.9 (11.2)	48.2 (10.4)	46.7 (10.6)
Age category, no. (%)			
18–44	4 (40)	4 (40)	4 (40)
45–59	6 (60)	3 (30)	5 (50)
≥60	0	3 (30)	1 (10)
Male, no. (%)	3 (30)	1 (10)	8 (80)
Female, no. (%)	7 (70)	9 (90)	2 (20)
Body mass, kg (SD)	74.3 (4.7)	76.7 (6.4)	80.7 (6)
Body-mass index, kg/m^2^ (SD)	25.9 (1.7)	26.9 (2.1)	26.4 (1.5)
Positive swab by RT- PCR, %	100	100	100
Duration of illness, days (SD)	3.4 (1.1)	2.7 (0.7)	3.4 (0.8)
≤7 days, no. (%)	10 (100)	10 (100)	10 (100)
>7 days, no. (%)	0	0	0
WHO Ordinal scale of clinical improvement (WHO-OSCI, score 0 to 8)			
Score 3, no. (%)	4 (40)	0	0
Score 4, no. (%)	6 (60)	10 (100)	10 (100)
Oxygen saturation, % (SD)	96.7 (1.1)	94.3 (0.7)	95.0 (0.9)
SpO_2_ ≥ 95%, no. (%)	10 (100)	6 (60)	6 (60)
SpO_2_ < 95%, no. (%)	0	4 (40)	4 (40)
Fever, °C (SD)	38.3 (0.1)	38.3 (0.3)	38.5 (0.4)
<37 °C, no. (%)	0	0	0
37–38 °C, no. (%)	0	1 (10)	1 (10)
>38 °C, no. (%)	10 (100)	9 (90)	9 (90)
Respiratory rate, min (SD) (normal range 16–20 min)	21.4 (1.6)	22.6 (0.7)	21.8 (1)
≤22 min, no. (%)	8 (80)	5 (50)	7 (70)
>22 min, no (%)	2 (20)	5 (50)	3 (30)
CRP, mg/L (SD) (normal < 5 mg/L)	21.5 (8.2)	38.9 (8.1)	37.8 (6.7)
D-dimer, ng/mL (SD) (normal < 243 ng/mL)	525.4 (175.7)	820.1 (133.1)	855.5 (142.5)
Neutrophil count × 10^9^ cells per L, no. (SD) (normal range 1.8–6.5)	3.0 (0.5)	2.2 (0.4)	2.7 (1.0)
Leukocyte count × 10^9^ cells per L, no. (SD) (normal range 3.2–10.6)	4.4 (0.7)	3.4 (0.4)	3.9 (1.5)
INR *, no. (SD) (normal range 0.85–1.15)	1.0 (0.2)	1.0 (0.1)	1.1 (0.1)
Prothrombin, % (SD) (quick test, normal range 95–105%)	103.3 (8.1)	78.5 (5.7)	78.2 (8.7)
Fibrinogen, g/L (SD) (normal range 2–4)	9.8 (2.6)	5.0 (1.2)	5.1 (2.2)
Involvement of the lung parenchyma, % (SD)	28.3 (7.6)	20.6 (6.8)	21.8 (6.1)
Chest CT 1 (< 25% abnormality), no. (%)	4 (40)	3 (30)	6 (60)
Chest CT 2 (25% –50% abnormality), no. (%)	6 (60)	7 (70)	4 (40)

* INR, International Normalized Ratio or prothrombin time.

Exclusion criteria were the following: refusal of the patient to participate; patients with respiratory rates >35 per min that did not decrease after their body temperature dropped to normal or sub-febrile values; blood oxygen saturation ≤93% at rest; partial pressure of oxygen in arterial blood (SpO_2_) <60 mm Hg; oxygenation index, SpO_2_ per fraction of inspired oxygen (SpO_2_/FiO_2_) ≤200 mm Hg; septic shock; chronic liver and kidneys diseases in terminal stage; refusal of other organs requiring control and treatment in the ICU; patients with HIV; using aprotinin within 6 months prior to screening; hypersensitivity to any of the components of the study therapy; patients participating in other clinical trials or taking other investigational drugs within 28 days of screening; pregnant or lactating women or women planning a pregnancy during the clinical study; women capable of childbirth who do not use adequate methods of contraception; patients unable to read or write or unwilling to understand and follow research protocol procedures; noncompliance with the regimen of taking medications or performing procedures, which, in the opinion of the investigator, may affect the results of the study or the safety of the patient and prevented the patient’s further participation in the study; and patients with any other comorbid medical or serious mental health conditions that rendered them ineligible for participation in clinical research, limited their ability to obtain informed consent, or affected their ability to participate in the research.
